# Case of Extra-Uterine Pregnancy

**Published:** 1873-08

**Authors:** W. Ross Jordan


### Case of Extra-Uterine Pregnancy. Gastrotomy successfully performed.

By W. Ross Jordan.

The woman aged twenty-nine, was a patient in the Birmingham Hospital for Women. In April last she had inflammation of the bowels, which threatened her life. In July or August she first felt the child, and in September she expected and prepared for her confinement. From this time she for six weeks gradually became smaller in size, after which she fancied she was in labour, being in great pain for three or four days. After that she had frequent shivers and a cold sensation in the abdomen. On the 13th December a swelling in the abdomen not larger than in ordinary pregnancy at six months was discovered, fluctuating a little towards the left side, and on deeper examination a round mass like the placenta between the umbilicus and pubis and a harder projection to the upper and left border to the tumour. The cervix uteri was pushed up to the right side. The sound penetrating three and a half inches pointed to the right groin and moved the round body felt in the abdominal examination. The recto-vaginal pouch was occupied by a hard rounded mass. On December 21st a puncture with the aspirator was decided upon, and a quantity of chocolate-coloured fluid mixed with white flakes was drawn. Mr. Ross Jordan, from his examination on this occasion, came to the conclusion that the case was one of extra-uterine fetation. Two hours after complete collapse came on, and hemorrhage into the cyst or abdomen was suspected. Five hours after the use of the aspirator an incision four inches long was made in the abdominal wall down to the peritoneum, when the cyst with the placenta under it presented. A clot of blood having been removed, the cyst, with a foot near the external opening, was drawn forward, but the wall of the cyst being thin, it ruptured, and through this opening the fetus was extracted. The placenta was left undisturbed, and the openings of the cyst and the abdominal wall were brought together by sutures of carbolized catgut, leaving an open wound about two and a half inches long, which was covered with a layer of tenax, &c. The patient progressed favorably, and on the 1st and 2nd of January large fragments of placenta were discharged, and on the 10th of April she came to the hospital looking well with the wound quite closed.—*Obstetrical Journal Gt. Britain and Ireland.*

